# Initiating Psychotropic Treatment in a Patient With Alpha-Gal Syndrome

**DOI:** 10.7759/cureus.28443

**Published:** 2022-08-26

**Authors:** Matthew R Narlesky, Angelica Palting, Suporn Sukpraprut-Braaten, Andrew Powell, Robert Strayhan

**Affiliations:** 1 Psychiatry, Unity Health - White County Medical Center, Searcy, USA; 2 Research, Unity Health - White County Medical Center, Searcy, USA; 3 Research, Arkansas College of Osteopathic Medicine, Fort Smith, USA

**Keywords:** ssri, tick-borne, tick bite, amblyomma americanum, meat allergy, oligosaccharide, lone star tick, alpha-gal syndrome, alpha-gal allergy, alpha-gal

## Abstract

Alpha-gal syndrome, which is typically acquired by a tick bite, is an IgE-mediated immune response to galactose-alpha-1,3-galactose (alpha-gal), an oligosaccharide in most mammalian tissue. This report describes a 29-year-old Caucasian female with comorbid alpha-gal syndrome who presented to the inpatient psychiatric unit after an intentional overdose. Because of the patient’s alpha-gal syndrome, the treatment team worked with the hospital pharmacy to evaluate treatment options that did not contain mammalian products. After carefully reviewing the ingredients of suitable medications on formulary, the patient was started on a generic sertraline formulation that was free of mammalian derivatives. At the time of discharge, the patient reported significant symptom improvement and was free of symptoms suggesting an alpha-gal allergic reaction. This case illustrates the challenges of starting psychiatric medications in a patient with comorbid alpha-gal syndrome.

## Introduction

Alpha-gal syndrome is a condition in which individuals develop an allergy to mammalian products [1]. The syndrome is caused by an Immunoglobulin E (IgE) response to galactose-alpha-1,3-galactose (alpha-gal), an oligosaccharide found in all mammals except humans and other catarrhine primates, such as gorillas and chimpanzees [1,2]. Alpha-gal syndrome was discovered when researchers noticed a trend of post-infusion anaphylaxis in patients treated with cetuximab, a monoclonal antibody used to treat colorectal cancer and squamous cell carcinoma [3]. Researchers identified an association between patients experiencing post-infusion anaphylaxis and pre-existing IgE antibodies toward the alpha-gal epitope on the fragment antigen-bind portion of the cetuximab molecule [3]. Further research linked the primary sensitization to alpha-gal with bites from the lone star tick (*Amblyomma americanum*), a tick native to the southern, eastern, and central parts of the United States [4]. Since its discovery, alpha-gal syndrome has been identified in Australia, Europe, Central and South America, Asia, and Africa [5].

Diagnosing alpha-gal syndrome begins with a thorough clinical history that includes the specifics of the allergic reaction, timing of symptom onset, and severity of reaction [6]. The hallmark symptom of alpha-gal syndrome is a delayed allergic reaction that typically occurs 3-6 hours after exposure to alpha-gal [1]. The severity of the allergic reaction ranges from urticaria and angioedema to anaphylaxis [1,7]. Diagnosis of alpha-gal syndrome is complicated by the intermittent nature of symptom presentation, which has been hypothesized to be related to variations in the amount of allergen and fat content ingested [7]. Several tests are used in the diagnosis of alpha-gal syndrome. Skin prick tests with commercial extracts and fresh meat are generally viewed as unreliable [1]. Intradermal testing yields more robust results but is not practical for routine testing [5]. One of the more reliable testing methods is the ImmunoCAP IgE assay, which measures serum IgE to alpha-gal [1]. The gold standard for diagnosing alpha-gal syndrome is a food challenge [5,6].

There are several differential diagnoses that should be considered when evaluating the possibility of alpha-gal syndrome. One of the primary differential diagnoses is pork-cat syndrome. In this cross-reactive syndrome, IgE toward cat albumin reacts with porcine albumin, resulting in a severe allergic reaction when pork is ingested [7]. The allergic reaction ranges from pruritus to anaphylaxis, but unlike the allergic reaction in alpha-gal syndrome, the allergic response is not delayed [7]. Patients with pork-cat syndrome will have IgE positivity to both pork and cat serum albumin but will lack IgE antibodies to alpha-gal [6]. Beef-milk syndrome should also be considered as a differential diagnosis, especially in children. This syndrome is caused by IgE toward bovine serum albumin [6].

Alpha-gal syndrome treatment centers on avoiding exposure to the alpha-gal oligosaccharide [5]. Patients should supplement with iron and vitamin B12 to avoid deficiencies. Treatment of an allergic reaction involves the use of oral antihistamines in mild cases and intramuscular epinephrine in severe presentations [6]. Unfortunately, alpha-gal is common in medications because mammalian products, such as magnesium stearate, glycerin, and lactic acid, are often used as inactive ingredients [2]. Lactose alone is used in approximately 45 percent of all tablets and capsules in the United States [8]. Examples of common generic medications containing mammalian products include hydromorphone, pregabalin, buspirone, and oxycodone, among many others [2]. In this case, we describe the challenges of initiating psychotropic treatment in a patient with alpha-gal syndrome.

## Case presentation

This report describes the case of a 29-year-old Caucasian female, with a past medical history significant for alpha-gal syndrome, renal stones, vitamin D deficiency, and anemia, who was admitted to the inpatient psychiatric unit after an intentional overdose with an aspirin/acetaminophen/caffeine combination drug. Five months prior to her admission, she had been diagnosed with alpha-gal syndrome after her primary care provider ordered a celiac panel to evaluate abdominal discomfort, diarrhea, and fatigue. The celiac panel was significant for an alpha-gal IgE antibody level of 0.25 kUa/L (reference value for normal is less than 0.10 kUa/L); the remainder of her celiac panel was unremarkable.

After medical clearance by the emergency department, the patient was admitted to the inpatient psychiatric unit. On the day of admission, the patient presented with anhedonia, depressed mood, suicidality, poor concentration, anxiety, insomnia, decreased energy, diminished appetite, flashbacks, and nightmares. She did not have a history of an obvious hypomanic or manic episode. She reported several psychosocial stressors, including a recent divorce, financial difficulties, and struggling to care for her two young children. She had been diagnosed with posttraumatic stress disorder and major depressive disorder in the past. Her psychiatric family history was significant for bipolar disorder in her father. She reported a history of several hospitalizations for depression and suicidality. Her trauma history was significant for physical and sexual abuse. She was not on any psychotropics at the time of admission, although she had an extensive history of treatment with psychotropics, including trials of gabapentin, escitalopram, paroxetine, venlafaxine, fluoxetine, aripiprazole, buspirone, hydroxyzine, lamotrigine, bupropion, trazodone, and risperidone, among others which she could not recall. She reported medications had been helpful for her symptoms in the past, but she had discontinued them because she did not feel she needed them anymore. Of note, she had not been on any psychotropics since being diagnosed with alpha-gal syndrome. The patient had a Patient Health Questionnaire-9 of 22 at the time of admission; her vitals and labs were unremarkable.

The patient was provisionally diagnosed with posttraumatic stress disorder; major depressive disorder, recurrent, severe; and adjustment disorder with disturbance of conduct. To address the patient’s presenting symptoms, the treatment team opted to initiate treatment with a selective serotonin reuptake inhibitor. Escitalopram and sertraline were the primary considerations because of minimal drug interactions and suitability for treating the patient’s symptoms. The treatment team collaborated with the hospital pharmacy to evaluate treatment options that did not contain mammalian derivatives. As there were several different manufacturers for both medications, the national drug code was used to ensure that the listed ingredients were associated with the correct generic formulation. After ensuring the medication was free of mammalian derivatives, the patient was started on sertraline manufactured by Cipla USA at a dose of 50 mg by mouth daily. In addition to pharmacologic treatment, the patient participated in milieu programming and group therapy.

On the second day of her hospitalization, the patient reported continued depressed mood, anxiety, and insomnia. She had received her first dose of sertraline and did not exhibit urticaria, pruritus, or other symptoms suggestive of an alpha-gal allergic reaction. The patient reported a reduction in her depression and suicidal ideation on her third day of hospitalization. The patient’s sertraline dose was increased to 100 mg by mouth daily to better address her depressive symptoms. The patient participated in a family meeting with her parents and the social worker. On days four and five of her hospitalization, the patient reported continued improvement in her depression and felt she could better manage her anxiety with her coping skills.

On the sixth day of her hospitalization, having demonstrated sustainable symptom improvement, the patient was discharged to her home. At the time of discharge, the patient was medically stable, compliant with her medications, and free of suicidal ideation. She reported a commitment to continuing treatment with a psychiatrist and therapist in the outpatient setting. She continued to be free of symptoms that would suggest an allergic reaction to alpha-gal. The patient was discharged with a 30-day prescription for sertraline 100 mg by mouth daily and appointments for therapy and medication management.

At her psychiatry appointment six weeks after discharge, the patient reported that her symptoms continued to be adequately managed by sertraline. She was regularly attending therapy and had enrolled in college classes. Fourteen weeks after her discharge, she presented to her allergist for a follow-up of alpha-gal syndrome. She had a skin test for raw meats and did not exhibit an allergic response. Her allergist said she could try small amounts of meat and if tolerated could continue to eat small amounts.

## Discussion

This case illustrates the challenges of initiating treatment with psychotropics in a patient with alpha-gal syndrome. One of the barriers to adequate treatment for patients with comorbid alpha-gal syndrome is the lack of awareness in the medical community. This was highlighted in a study by Flaherty et al. indicating that three-quarters of surveyed alpha-gal syndrome patients determined they had alpha-gal syndrome with resources outside of medical care [9]. Furthermore, the majority of the surveyed patients rated their primary care providers as having little to no knowledge of the illness [9]. Increased awareness of alpha-gal syndrome would not only lead to earlier diagnosis but would also increase opportunities for providers to educate patients about lifestyle modifications that can have a significant impact on symptom severity. For instance, in addition to counseling on avoiding mammalian products, providers can educate patients on the role of cofactors, such as alcohol use, that have been shown to increase the severity of allergic reactions to alpha-gal [10]. More clinical awareness is especially important for providers who treat at-risk populations, such as hunters or forest workers [11,12]. Additionally, as there are significant geographic differences in alpha-gal syndrome prevalence, further research should evaluate the value of screening protocols in high-risk areas [13].

This case also highlights the necessity of interdisciplinary collaboration when treating psychiatric patients with comorbid alpha-gal syndrome. In this case report, the treatment team collaborated with the hospital pharmacy to evaluate treatment options that were free of mammalian derivatives. Coordination of care with the patient’s primary care provider and allergist was also important for characterizing the severity of the patient’s illness and ensuring adequate precautions were in place in the event of an allergic reaction. Initiating treatment with psychotropics in patients with comorbid alpha-gal syndrome is complicated by the ubiquity of mammalian products as inactive ingredients in medications. Moreover, some common pharmaceutical ingredients can be sourced from animals or plants [2]. For instance, stearic acid and lactic acid are generally alpha-gal positive if sourced from animals and alpha-gal negative if sourced from plants. Unfortunately, the sourcing information for these ingredients is not readily available in medication package inserts [2]. In the case described in this report, the pharmacy team had to review ingredients in each medication formulation to determine whether they contained ingredients derived from mammalian sources. Currently, pharmaceutical companies are not required to list the presence of alpha-gal content or the mass of excipients, which would be helpful for assessing potential tolerability in particularly sensitive patients [8,14]. As generic manufacturers often use different ingredients for the same medication, it is not feasible to generalize the alpha-gal content of one drug formulation to other formulations. Fortunately, the Food and Drug Administration (FDA) has taken steps to provide more information to the public about inactive ingredients in medications with the Inactive Ingredient Database [15]. Future research should evaluate strategies, such as a database of ingredients that contain alpha-gal, to streamline the process of selecting medications suitable for patients with comorbid alpha-gal syndrome.

Finally, this case report demonstrates the need for establishing clear guidelines for the diagnosis of alpha-gal syndrome. There is significant variability in the literature on IgE levels diagnostic of alpha-gal syndrome with published values ranging from ≥ 0.54 kUa/L to ≥ 10 kUa/L [1,11,12]. In the case described in this report, the patient’s alpha-gal IgE level was 0.25 kUa/L, which could be considered equivocal by some standards. The diagnostic and prognostic value of measuring IgE levels is, in and of itself, not well-characterized because of the poor correlation between IgE levels and symptom severity [5,6,12]. The relation between IgE levels and symptoms is further obfuscated by the apparent reduction in antibody levels over time if affected individuals avoid subsequent tick exposure [5,12]. Fortunately, there have been some developments in diagnostic tests, such as the basophil activation test, that appear promising [16]. As cases of alpha-gal syndrome continue to rise, established diagnostic and treatment guidelines will become increasingly important [5,14].

## Conclusions

This report describes the challenges of initiating psychotropic treatment in a patient with comorbid alpha-gal syndrome. Interprofessional collaboration is essential for providing patients with appropriate care. Increased awareness of alpha-gal syndrome in the medical community would ensure patients receive timely diagnosis and treatment. Given the complexity of identifying medications free of mammalian derivatives, there is a need for the development of strategies to streamline the process of selecting medications suitable for patients with alpha-gal syndrome. Despite advancements in research on alpha-gal syndrome, there is still significant room for further understanding of this illness.
